# National Assessment of Capacity in Public Health, Environmental, and Agricultural Laboratories — United States, 2011

**Published:** 2013-03-08

**Authors:** Matthew L. Boulton, Angela J. Beck, John M. DeBoy, Deborah H. Kim

**Affiliations:** Center of Excellence in Public Health Workforce Studies, Univ of Michigan School of Public Health; Maryland Dept of Health and Mental Hygiene; Association of Public Health Laboratories, District of Columbia

In 2011, the University of Michigan’s Center of Excellence in Public Health Workforce Studies and the Association of Public Health Laboratories (APHL) assessed the workforce and program capacity in U.S. public health, environmental, and agricultural laboratories (1). During April–August 2011, APHL sent a web-based questionnaire to 105 public health, environmental, and agricultural laboratory directors comprising all 50 state public health laboratories, 41 local public health laboratories, eight environmental laboratories, and six agricultural laboratories. This report summarizes the results of the assessment, which inquired about laboratory capacity, including total number of laboratorians by occupational classification and self-assessed ability to carry out functions in 19 different laboratory program areas. The majority of laboratorians (74%) possessed a bachelor’s degree, associate’s degree, or a high school education or equivalency; 59% of all laboratorians were classified as laboratory scientists. The greatest percentage of laboratories reported no, minimal, or partial program capacity in toxicology (45%), agricultural microbiology (54%), agricultural chemistry (50%), and education and training for their employees (51%). Nearly 50% of laboratories anticipated that more than 15% of their workforce would retire, resign, or be released within 5 years, lower than the anticipated retirement eligibility rate of 27% projected for state public health workers (2). However, APHL and partners in local, state, and federal public health should collaborate to address gaps in laboratory capacity and rebuild the workforce pipeline to ensure an adequate future supply of public health laboratorians.

The main objectives of the National Laboratory Capacity Assessment were to count and characterize the public health, environmental, and agricultural laboratory workforce, measure laboratory program area capacity, and assess worker recruitment, retention, and retirement plans. Information was collected on laboratory type, overall equipment quality, and number of workers in nine different job classifications by degree type: aide/assistant, technician, scientist, scientist-supervisor, scientist-manager, developmental scientist, environmental or agricultural laboratory director/assistant director, public health laboratory assistant/deputy director, and public health laboratory director. Questions evaluated capacity in 13 technical/scientific program areas: agricultural chemistry, agricultural microbiology, bacteriology, clinical chemistry/hematology, environmental microbiology, environmental chemistry, molecular biology, mycology, newborn screening, parasitology, serology/immunology, and virology, and in six administrative program areas, including emergency preparedness and response, education and training, quality assurance, regulation and inspection, safety and/or security, and laboratory administration/operations. Additional questions focused on worker recruitment, retention, and planned releases, retirements, and resignations.

The organizational survey was pilot tested in four states and included interviews with all pilot testers; the assessment was available online to all states during July 1–August 30, 2011. The director of the state public health, environmental, or agricultural laboratory was the designated key informant. In follow-up, APHL contacted laboratory directors by e-mail and telephone to maximize response. Eighty (76%) of 105 laboratory directors participated. A laboratorian was defined as a person whose principal work was in a governmental public health, environmental, or agricultural laboratory; positions could be reported as one-quarter fractions of full-time equivalents. For each program area, the director was asked if they had adequate capacity to perform necessary services for that program area. Estimates were categorized as follows: full = 100% capacity to perform; almost full = 75%–99%; substantial = 50%–74%; partial = 25%–49%; minimal = 1%–24%; and none. Fifty-six (71%) of the laboratories self-identified as a public health (49), environmental (three), or agricultural (four) laboratory; 23 (29%) self-identified as some combination of these categories; and one did not specify laboratory type.

In 2011, a total of 6,656 employees, of whom 5,555 (83%) were laboratorians in one of the eight job classifications identified, worked in the 80 responding laboratories; the remaining 894 employees (13%) were administrative support staff, and 207 (4%) were information technology staff (Table 1). The distribution of full-time equivalents in 19 different laboratory program areas showed the greatest number of employees working in environmental chemistry (780 [14%]), followed by bacteriology (558 [10%]), administration/operations (533 [10%]), newborn screening (514 [9%]), other (480 [9%]), emergency preparedness and response (414 [7%]); laboratory regulation and inspection (343 [6%]), and serology/immunology (325 [6%]), with 5% or fewer of employees working in each of the remaining 12 laboratory program areas. Education and training background was provided for 4,927 employees by position (Table 2). Of these, 587 (12%) had a doctoral or professional degree, 701 (14%) had a master’s degree, 3,249 (66%) had a bachelor’s or associate’s degree, and 390 (8%) had a high school education or equivalency. Laboratory scientists were the largest group (59%), with 13% scientist-supervisors, 9% technicians, 7% aides or assistants, 6% scientist-managers, 3% developmental scientists, 2% directors, and 1% deputy or assistant directors. On average, 38% of laboratory employees were supported by state funding, 21% by local sources, 20% by federal funds, 19% by fee-for-service, and 2% by other sources.

For those laboratories indicating any capacity in a given program area (i.e., a response other than “not applicable”), more than half reported either no, minimal, or only partial capacity to perform necessary activities in toxicology (65%), agricultural chemistry (80%), agricultural microbiology (80%), clinical chemistry/hematology (68%), and education and training (55%). Conversely, more than 75% of laboratories reported substantial to full capacity in emergency preparedness and response (89%), safety and/or security (91%), bacteriology (91%), administration/operations (86%), molecular biology (92%), quality assurance (83%), serology/immunology (85%), and regulation and inspection (80%); however, fewer than half reported substantial to full capacity in agricultural chemistry (20%), agricultural microbiology (20%), clinical chemistry/hematology (32%), toxicology (35%), and education and training (45%) (Figure). Several program areas with lower capacity also were the same ones for which approximately one third or more laboratories selected “not applicable,” including clinical chemistry/hematology (41%), agricultural chemistry (38%), agricultural microbiology (33%), and toxicology (31%). Fifty-one percent of laboratories reported the overall quality of their equipment and instrumentation on a 5-point scale as fair, the remainder as good or very good.

More than half of laboratories (42 [53%]) anticipated that up to 15% of their workers would retire, resign, or be released within 5 years, whereas 27 (34%) laboratories predicted a loss of 16%–25%, 10 (13%) predicted a loss of 26%–50%, and one anticipated losing more than 75% within 5 years. The lack of opportunities for promotion and lack of a career path for advancement were the two most common barriers to recruitment; both were reported by 76% of responding laboratories. Other major barriers to recruitment were inadequate salary scale (59 [74%]), hiring policies/procedures (54 [68%]), and complexity of the administrative bureaucracy (49 [61%]). Lack of promotion opportunities and career path for advancement were reported as obstacles to worker retention by 66 (83%) and 64 (80%) of laboratories, respectively, as was inadequate salary scale by 58 (73%) of laboratories.

## Editorial Note

The public health, environmental, and agricultural laboratory workforce is a vital component of the nation’s public health infrastructure. Laboratory capacity is essential for protection against health hazards and provision of essential community services (3). The 2011 National Laboratory Capacity Assessment revealed that laboratories have low capacity in several key program areas, especially in agriculture-related services, toxicology, and in the general area of worker training; laboratories also were much more likely to select “not applicable” regarding their capacity in the areas of clinical chemistry/hematology, agricultural chemistry, agricultural microbiology, and toxicology, indicating that testing and services in these areas are not provided. Given the growing importance of the human-animal interface in the risk for emerging disease threats (4), the gaps in agricultural microbiology and chemistry should be addressed, and opportunities for creating stronger links among public health, agricultural, and veterinary laboratories should be explored. Dealing with low reported capacity in toxicology is equally imperative because environmental pollution and human exposures, such as pharmaceuticals in drinking water, are expected to increase (5), and the nation’s ability to quickly respond to unintentional and intentional chemical releases represents a core component of its preparedness capacity.

The lack of many laboratories’ ability to provide training to their staff is a concern because access to continuing education is essential to ensuring a well-trained public health workforce. This might be particularly true for laboratorians because only one quarter of laboratorians have a graduate or professional degree. Laboratory directors and public health, environmental, and agricultural laboratory workers reported lack of opportunities for promotion and a clear career path to advancement as the most common barriers to worker recruitment and retention (1), which might be related to the limited number of educational and training opportunities available to laboratorians because of funding or other restrictions. This poses special challenges in the context of the findings on the projected laboratory workforce losses through planned resignation, release, and retirement. A need exists to increase the number and type of laboratory science degree and other training offerings in schools and programs of public health to successfully build the worker pipeline, especially given the severe and continuing shortage of scientists qualified to assume leadership and management positions within public health, environmental, and agricultural laboratories, a concern noted by APHL since 2006 (6). This could include training in laboratory leadership because currently no academic doctoral program in public health laboratory science and practice exists at any school of public health nationwide.

The assessment of laboratories’ program area capacity should be based on the Laboratory System Improvement Plan (LSIP) standards, which were developed by APHL and CDC to assess laboratory performance (7,8). However, very few laboratories completed the section of the survey concerning LSIP, which might indicate that they are not using the standards, are not familiar with them, or do not know how to evaluate their use. Additional marketing and educational efforts should be directed at increasing awareness and encouraging use of the LSIP.

The findings of this report are subject to at least three limitations. First, only three quarters of laboratory directors completed the survey; nonresponders might have differed systematically from responders and yielded dissimilar results if they had participated. Second, the questions used to assess program area capacity in the laboratory were subjective, and their interpretation might have varied by respondent. Finally, some subjective questions have objective correlates, such as assessment of equipment quality.

What is already known on this topic?

The public health, environmental, and agricultural laboratory workforce is a vital component of the nation’s public health infrastructure. Well-trained laboratorians are essential to providing protection against newly emergent diseases and other health hazards through diagnostic testing; reporting and surveillance; chemical, toxicologic, and environmental analysis; emergency preparedness; and provision of other vital services for the community.

What is added by this report?

Data from a 2011 National Laboratory Capacity Assessment indicate that national public health laboratory capacity needs to improve in several areas to achieve optimal testing and response capacity. Laboratory workers need better access to training and educational opportunities to ensure a well- qualified laboratory workforce.

What are the implications for public health practice?

Agencies at the local, state, and federal level should collaborate to improve laboratory capacity, including worker training and education, and encourage the development of a greater number and type of available laboratory degree programs.

The *Healthy People 2020* public health infrastructure objective no. 11 (PHI-11) aims to increase the proportion of tribal and state public health agencies that provide or ensure comprehensive laboratory services to support essential public health services (9). The National Laboratory Capacity Assessment described in this report represents an initial attempt to measure baseline national capacity in public health, environmental, and agricultural laboratories and should be repeated in the future.

## Figures and Tables

**FIGURE:**
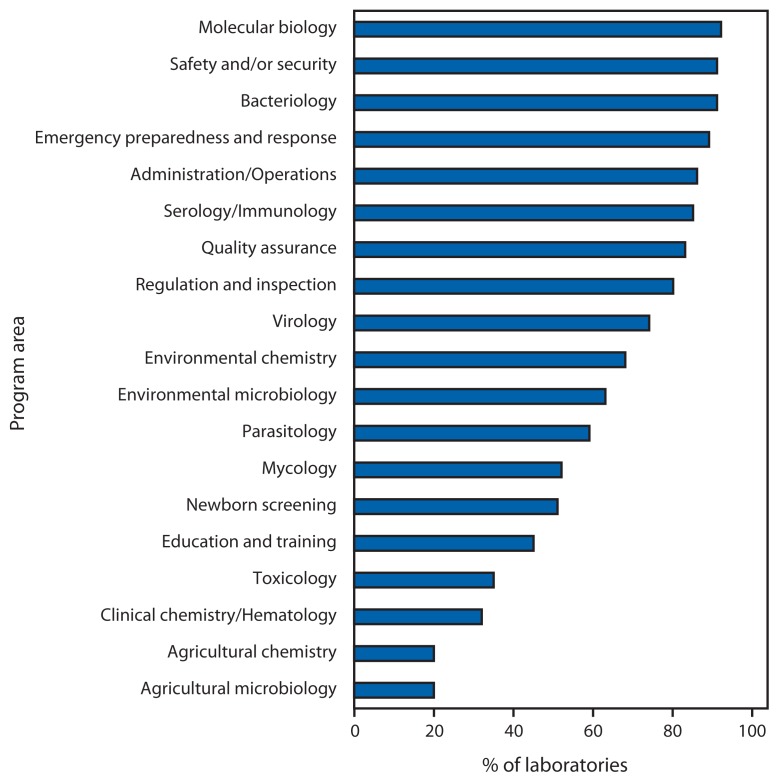
Percentage of laboratories (N = 80) reporting substantial to full capacity, by program area — National Laboratory Capacity Assessment, United States, 2011

**TABLE 1:** Number and percentage of administrative, information technology, and scientific staff in laboratories, by program area — National Laboratory Capacity Assessment, United States, 2011

Program area	No.	(%)
**Administrative staff**	**894**	**(13)**
**Information technology staff**	**207**	**(3)**
**Scientific laboratory staff**	**5,555**	**(83)**
Agricultural chemistry	133	(2)
Agricultural microbiology	74	(1)
Bacteriology	558	(10)
Clinical chemistry/Hematology	70	(1)
Education and training	84	(2)
Emergency preparedness and response	414	(7)
Environmental microbiology	241	(4)
Environmental chemistry	780	(14)
Administration/Operations	533	(10)
Quality assurance	127	(2)
Regulation and inspection	343	(6)
Safety and/or security	64	(1)
Molecular biology	286	(5)
Mycology	48	(1)
Newborn screening	514	(9)
Parasitology	61	(1)
Serology/Immunology	325	(6)
Toxicology	136	(2)
Virology	284	(5)
Other	480	(9)
**Total laboratory staff**	**6,656**	

**TABLE 2:** Number and percentage of laboratory workers, by position and degree type — National Laboratory Capacity Assessment, United States, 2011

Degree	Aide/Assistant	Technician	Scientist	Scientist-supervisor	Scientist-manager	Developmental scientist	Assistant/Deputy director	Director	Total	

									No.	(%)
Doctoral degree	1	1	168	67	120	81	30	61	**529**	**(11)**
Professional degree*	1	2	21	13	4	3	2	12	**58**	**(1)**
Master’s degree	5	9	399	143	83	17	23	22	**701**	**(14)**
Bachelor’s degree	70	167	2,177	416	94	31	14	5	**2,974**	**(60)**
Associate’s degree	44	120	94	14	1	0	0	2	**275**	**(6)**
High school or equivalent	217	138	26	7	1	0	0	1	**390**	**(8)**
**Total**	**338 (7)**	**437 (9)**	**2,885 (59)**	**660 (13)**	**303 (6)**	**132 (3)**	**69 (1)**	**103 (2)**	**4,927** †	**(100)**

* E.g., MD, DVM, and DDS.

† Position and degree information was not reported for 628 laboratory workers.
